# Multimodal deep learning for predicting PD-L1 biomarker and clinical immunotherapy outcomes of esophageal cancer

**DOI:** 10.3389/fimmu.2025.1540013

**Published:** 2025-03-11

**Authors:** Hui Liu, Yinpu Bai, Zhidong Wang, Shi Yin, Cheng Gong, Bin Wang

**Affiliations:** ^1^ College of Computer and Information Engineering, Nanjing Tech University, Nanjing, Jiangsu, China; ^2^ Department of Cardiothoracic Surgery, the Third Affiliated Hospital of Soochow University, Changzhou, Jiangsu, China

**Keywords:** esophageal squamous cell carcinoma, PD-L1 biomarker, multi-modal deep learning, immunotherapy response, pathology image, CT imaging

## Abstract

Although the immune checkpoint inhibitors (ICIs) have demonstrated remarkable anti-tumor efficacy in solid tumors, the proportion of ESCC patients who benefit from ICIs remains limited. Current biomarkers have assisted in identifying potential responders to immunotherapy, yet they all have inherent limitations. In this study, two ESCC cohorts were established from the Third Affiliated Hospital of Soochow University in China. One cohort included 220 patients with PD-L1 expression levels determined by immunohistochemistry, and the other cohort included 75 patients who underwent immunotherapy. For each patient in both cohorts, we curated multimodal data encompassing Hematoxylin and Eosin-stained pathology images, longitudinal computed tomography (CT) scans, and pertinent clinical variables. Next, we introduced a novel multimodal deep learning model that integrated pathological features, radiomic features, and clinical information to predict PD-L1 levels, immunotherapy response, and overall survival. Our model achieved an AUC value of 0.836 for PD-L1 biomarker prediction, and 0.809 for immunotherapy response prediction. Furthermore, our model also achieved an AUC value of 0.8 in predicting overall survival beyond one or three years. Our findings confirmed that the multimodal integration of pathological, radiomic, and clinical features offers a powerful means to predict PD-L1 biomarker levels and immunotherapy response in esophageal cancer.

## 1 Introduction

Esophageal cancer is one of the most prevalent cancers and a leading cause of cancer-related mortality worldwide (1), accounting for more than 500,000 deaths each year (2). Esophageal squamous cell carcinoma (ESCC) is the main histological subtype that has distinct epidemiological and clinical characteristics. It is prone to lymphatic spread and associated with poor prognosis (3). Immune checkpoint inhibitors (ICIs) have demonstrated remarkable anti-tumor efficacy in ESCC patients. However, the proportion of ESCC patients who benefit from ICIs remains limited.

In current clinical practice, the choice of immunotherapy is mainly guided by the levels of PD-L1 biomarker within tumor tissue (4). Elevated levels of PD-L1 are often indicative of favorable response to immunotherapy (5). Nonetheless, the assessment of PD-L1 biomarker via immunohistochemistry (IHC), commonly quantified as the combined positive score (CPS) or tumor proportion score (TPS) (6), is both resource-intensive and time-consuming (7). The variability in PD-L1 quantification is also substantial, influenced by the staining method and antibody employed (8). Some studies have reported a low rate of reproducibility for PD-L1 assessment by certified pathologists (9, 10). Besides, its nontrivial interpretation, coupled with the absence of a universal expert consensus, exacerbates the challenges in clinical decision-making (11).

Hematoxylin and eosin (H&E) staining is a routine examination of clinical specimens, facilitating the visual inspection of malignant cells (12). Pathologists rely on H&E staining for tumor diagnosis, including the determination of tumor subtype and grade. Recent advancements in computational pathology have achieved performance on par with that of pathologists in tasks such as tumor diagnosis and grade classification (13). Deep learning methodologies can capture information from H&E images beyond human visual capability, thereby offering new potential for pathology slides. For instance, Shamai et al. demonstrated that the expression levels of molecular biomarkers could be predicted from H&E whole slide images of breast cancer (7). Jin et al. introduced a multiple instance learning method for pan-cancer PD-L1 level prediction from histopathology slides, highlighting its potential to identify diverse histological patterns indicative of molecular levels (14). Despite these advancements, there is currently no evidence supporting the use of H&E slide analysis for predicting PD-L1 levels in esophageal cancer.

Moreover, radiomic features derived from regions of interest (ROIs) of radiographic imaging, such as lesion shape, size, voxel intensity, and texture, have demonstrated strong correlations with transcriptional and protein expression of clinical biomarkers in solid tumors (4, 15–18). For instance, Tian et al. proposed a deep learning framework based on CT images to non-invasively assess PD-L1 expression and immunotherapy response in NSCLC patients (15). Mu et al. developed a residual deep network utilizing pre-treatment PET/CT images to predict PD-L1 expression, as well as the durable clinical benefit, progression-free survival (PFS), and overall survival (OS) in advanced-stage NSCLC patients (4). However, radiomic features have not yet been explored for predicting the immunotherapy response of ESCC patients.

Currently, ESCC cohorts containing both H&E-stained slides and CT images with corresponding PD-L1 levels remain limited. In this paper, we invested our effort to construct two multimodal datasets in this retrospective study: a PD-L1 cohort and an immunotherapy cohort. For each patient, we manually collected the H&E whole-slide images, pre-treatment and early on-treatment CT images, as well as clinical variables. With the joint efforts of expert pathologists and computerized tools designed for rapid annotation, we successfully annotated the H&E slides and regions of interest (ROIs) in CT images. We think the esophageal cancer cohorts with manually curated immunotherapy response and multimodal data are valuable to the biomedical community. Next, we introduced a multimodal deep learning model to predict PD-L1 biomarker level and immunotherapy response. For H&E-stained slides, we employed deep convolutional networks to extract pathological tissue features. For CT images, we extracted radiomic features from the ROIs. When combined with clinical variables, these features demonstrated high predictive power for both PD-L1 levels and immunotherapy outcomes.

## 2 Materials and methods

### 2.1 Ethical review approval

This study was approved by the Institutional Review Boards of the Third Affiliated Hospital of Soochow University (Approval number: 2024-KD139) and was performed in accordance with the Declaration of Helsinki.

### 2.2 Patient cohorts

For the task of PD-L1 level prediction, we retrospectively curated a PD-L1 cohort of ESCC patients who underwent esophagectomy. The inclusion criteria were: (a) histologically confirmed ESCC; (b) treatment by surgery; (c) availability of complete clinical records. The exclusion criteria were: (a) receipt of neoadjuvant therapy before surgery; (b) baseline contrast-enhanced chest CT images of poor quality or with unmeasurable lesions; (c) poor-quality H&E images. The clinical variables include: age, sex, BMI, smoking history, drinking history, hypertension, tumor node metastasis (TNM) stage, grade, neurovascular invasion, tumor size, adjuvant radiotherapy and chemotherapy. The clinical endpoint of interest was overall survival, defined as the time from the treatment of esophagectomy to death from any cause or the latest follow-up. The latest follow-up period ended on 1 January 2024. As a result, the PD-L1 cohort included 220 patients, and the detailed summary is presented in **Table 1**. The PD-L1 levels were examined by immunohistochemistry assay on formalin-fixed, paraffin-embedded samples, using PD-L1 22C3 antibody on the Dako Link 48 platform (RRID: AB 2889976). The PD-L1 levels were reported in the form of combined positive scores (CPS) that fall in [0–100]. Two pathologists independently evaluated the ESCC slides to determine the PD-L1 expression in both tumor cells and immune cells, including lymphocytes and macrophages.

**Table 1:** Demographic and clinical variables of ESCC PD-L1 cohort.

	ESCC PD-L1 cohort		
	All	PD-L1<50%	PD-L1≥ 50%
		(N=188)	(N=32)
Age (years)			
Mean ± SD	66.5 ± 8.1	66.7 ± 8.1	65.4 ± 7.0
Gender, n (%)			
Female	45 (20.5)	37 (19.7)	8 (25.0)
Male BMI	175 (79.5)	151 (80.3)	24 (75.0)
Mean ± SD	22.56 ± 3.31	22.53 ± 3.28	22.76 ± 3.51
Smoking history, n (%)			
No	127 (57.7)	109 (58.0)	18 (56.2)
Yes	93 (42.3)	79 (42.0)	14 (43.8)
Drinking history, n (%)			
No	136 (61.8)	115 (61.2)	21 (65.6)
Yes	84 (38.2)	73 (38.8)	11 (34.4)
Hypertension, n (%)			
No	124 (56.4)	106 (56.4)	18 (56.2)
Yes	96 (43.6)	82 (43.6)	14 (43.8)
TNM Stage, n (%)			
I	19 (8.6)	16 (8.5)	3 (9.4)
II	95 (43.2)	83 (44.1)	12 (37.5)
III	94 (42.7)	78 (41.5)	16 (50.0)
IV	12 (5.5)	11 (5.9)	1 (3.1)
Grade, n (%)			
G1	20 (9.1)	15 (8.0)	5 (15.6)
G2	139 (63.2)	124 (66.0)	15 (46.9)
G3	61 (27.7)	49 (26.1)	12 (37.5)
Neurovascular invasion, n (%)			
No	123 (55.9)	104 (55.3)	19 (59.4)
Yes	97 (44.1)	84 (44.7)	13 (40.6)
Tumor size			
Mean ± SD	12.31 ± 9.48	12.21 ± 9.76	12.89 ± 7.74
Adjuvant radiotherapy, n (%)			
No	135 (61.4)	117 (62.2)	18 (56.2)
Yes	85 (38.6)	71 (37.8)	14 (43.8)
Adjuvant chemotherapy, n (%)			
No	121 (55.0)	107 (56.9)	14 (43.8)
Yes	99 (45.0)	81 (43.1)	18 (56.2)
Overall survival (months)			
Median	24.65	24.43	26.03

For immunotherapy response prediction, we built an immunotherapy cohort of patients who received immune checkpoint inhibitors (Tislelizumab, Camrelizumab, and Sintilimab) alone or in combination with chemotherapy. The inclusion criteria were: (a) histologically confirmed ESCC via endoscope; (b) stage III/IV; (c) receipt of immunotherapy alone or in combination with chemotherapy; (d) availability of contrast-enhanced chest CT within 2 months before the start of immunotherapy; and (e) completion of 2-4 cycles of treatment with follow-up CT images available for response evaluation. The exclusion criteria were: (a) receipt of surgery or radiotherapy during immunotherapy; (b) incomplete clinical records; and (c) poor-quality CT and H&E images. Immunotherapy response was independently evaluated by two experienced radiologists and one oncologist according to RECIST version 1.1 (19, 20). The immunotherapy cohort included 75 patients, and the clinical variables are presented in **Table 2**. We also provided the clinical and radiomics features collected before treatment for both the PD-L1 and immunotherapy cohorts (**Supplementary Table S6**), and the radiomics features after treatment for the immunotherapy cohort (**Supplementary Table S7**).

**Table 2:** Demographic and clinical variables of ESCC immunotherapy response cohort.

	ESCC immunotherapy cohort		
	All	Non-responder	Responder
		(N=39)	(N=36)
Age (years)			
Mean ± SD	67.04 ± 8.56	68.23 ± 8.73	65.75 ± 8.31
Gender, n (%)			
Female	10 (13.33)	4 (10.26)	6 (16.67)
Male BMI	65 (86.67)	35 (89.74)	30 (83.33)
Mean ± SD	21.98 ± 2.88	21.93 ± 2.95	22.02 ± 2.86
Smoking history, n (%)			
No	49 (65.33)	28 (71.79)	21 (58.33)
Yes	26 (34.67)	11 (28.21)	15 (41.67)
Drinking history, n (%)			
No	55 (73.33)	30 (76.92)	25 (69.44)
Yes	20 (26.67)	9 (23.08)	11 (30.56)
Hypertension, n (%)			
No	49 (65.33)	27 (69.23)	22 (61.11)
Yes	26 (34.67)	12 (30.77)	14 (38.89)
TNM Stage, n (%)			
III	49 (65.33)	25 (64.10)	24 (66.67)
IV	26 (34.67)	14 (35.90)	12 (33.33)
Treatment strategy, n (%)			
Immunotherapy	4 (5.33)	4 (10.26)	0 (0.00)
Immunotherapy+Chemotherapy	71 (94.67)	35 (89.74)	36 (100.00)
PD-L1/PD-1 inhibitor, n (%)			
Sintilimab	59 (78.67)	30 (76.92)	29 (80.56)
Camrelizumab	10 (13.33)	4 (10.26)	6 (16.67)
Tislelizumab	6 (8.00)	5 (12.82)	1 (2.78)
Progression-free survival (months)			
Median	14.27	13.33	14.42
(95%CI)	(11.97-16.56)	(9.64-17.03)	(11.76-17.08)

### 2.3 Multimodal learning framework

**Figure 1:**
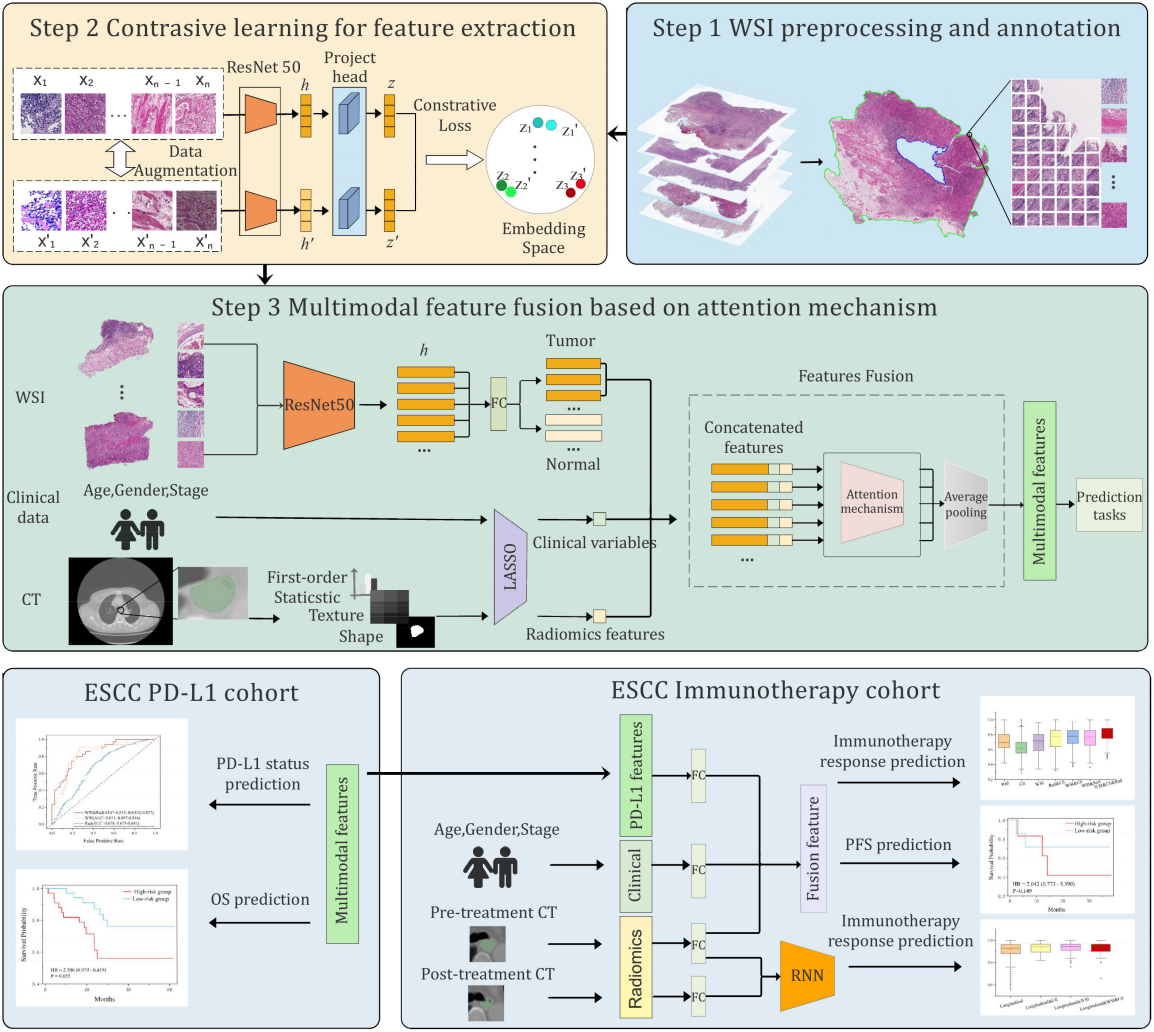
Overview of the proposed multimodal deep learning framework. The framework involved three steps: preprocessing and annotation of whole slide images (WSIs) and CT images, pretraining of a contrastive learning model to extract patch-level features, and integration of multimodal features by attention mechanism. The multimodal features were applied for PD-L1 level and immunotherapy response prediction, as well as prognosis.

Our learning framework leveraged self-supervised contrastive learning and multimodal fusion techniques. Unlike previous studies that utilized only WSIs or CT images, our method integrated multimodal features across multiple prediction tasks, thereby achieving better performance. Overall, the framework consisted of three stages (**Figure 1**). First, the WSIs were segmented and tessellated into patches, which were labeled as tumor or non-tumor based on pathologist annotations. All CT images were delineated to identify regions of interest (ROI), from which radiomic features were extracted. Second, we trained a contrastive learning model on a large number of unlabeled patches, thereby extracting expressive patch-level features for downstream tasks. Finally, we used LASSO to select important features from the radiomic features and clinical variables. These selected features were then aggregated with the pathological features of tumor patches through an attention mechanism. The multimodal features were used for downstream tasks, including PD-L1 level assessment, immunotherapy response prediction, and prognosis evaluation.

### 2.4 Data preprocessing and annotation

The preprocessing of WSIs involved tissue segmentation and tiling. For each slide, we used OpenSlide to read it into memory and then converted it from RGB to HSV color space (21). To identify tissue regions (foreground), a binary mask was generated by thresholding the saturation channel in HSV space. The edges were smoothed, and morphological closure was applied to fill small gaps and holes, effectively segmenting the slide into tissue and non-tissue regions. Following segmentation, each WSI was split into 256×256 pixel patches within the foreground region at 20x magnification. As a result, we obtained 742,978 patches, with an average of 3,377 patches per WSI.

Patches were filtered to exclude those with insufficient tissue content, using a threshold where pixel values greater than 210 were considered white. Two pathologists with more than 10 years of clinical experience independently annotated the tumor regions on each slide. Patches overlapping the annotated tumor regions were labeled as tumor, and non-tumor otherwise.

All preoperative contrast-enhanced chest CT images from both cohorts were independently reviewed by 2 cardiothoracic surgeons who had 10 years of clinical experience. They manually delineated the region of interest (ROI) using the open-source software 3D Slicer (https://www.slicer.org/) to perform image segmentation.

### 2.5 Contrastive learning for pathological feature extraction

Given a large number of unlabeled patches, we leveraged contrastive learning to train an encoder to extract the intrinsic features of each patch (22, 23). The main idea of contrastive learning is to bring positive samples close to each other in the latent space and push negative samples to be far apart, by setting a pretext task. In our practice, we generated two views of a patch via image augmentation as positive pairs, while the augmentations of other patches in a mini-batch were regarded as negative samples. We employed a diverse range of image augmentations, including random proportion cropping and scaling, random color jittering, random Gaussian blurring, and random flipping. The ResNet50 CNN network pre-trained on ImageNet (24) was used as the encoder backbone *f*, which yields a 1024-dimensional latent representation $h_{i}$ for an input patch $x_{i}$ , namely, $h_{i}=f\left(x_{i}\right)$ . Next, a projection head *g* transforms the latent representation into embedding $z_{i}$ . Formally, we have $z_{i}=g\left(h_{i}\right)$ , and the contrastive loss of the input patch $x_{i}$ is defined as:


$$L=-log\frac{exp\left(sim\left(z_{i},z_{i}^{'}\right)/\tau \right)}{exp\left(sim\left(z_{i},z_{i}^{'}\right)/\tau \right)+\sum_{j=1}^{N}exp\left(sim\left(z_{i},z_{j}\right)/\tau \right)}$$

where $z_{i}$ and $z_{i}^{'}$ represent the embeddings of a pair of positive samples regarding the input patch *x_i_*, *sim*() is the cosine similarity obtained by dot product of two embeddings after L2 normalization. *N* is the size of the negative sample queue, $z_{j}$ refers to the embedding of a negative sample and *τ* is the temperature parameter. The size and the quality of negative samples greatly affect the performance of contrastive learning. Inspired by our previous work (22), we adopted the adversarial contrastive learning method AdCo (25) to pre-train the encoder. It treats negative samples as learnable weights and alternately updates adversarial samples to generate the most challenging negative samples.

After the contrastive learning finished, the encoder was used to extract the patchlevel features. To extend the applicability of our model, we used the features of the labeled patches (tumor vs. non-tumor) to train a classifier with only one fullyconnected layer to identify tumor patches. The classifier achieved an AUC value of 0.903.

### 2.6 Multimodal feature fusion

Following the extraction of pathological features, we used the Python package PyRa-diomics (26) to extract radiomics features from the ROIs that were manually annotated by physicians. For each CT image, 118 features were extracted, including first-order statistical features, as well as shape and texture features. The clinical variables listed in **Tables 1** and **2** were also taken into account.

Next, we employed the Least Absolute Shrinkage and Selection Operator (LASSO) algorithm (27) to select important radiomic and clinical features for prediction tasks. The selected features were then concatenated with the pathological features of tumor patches, resulting in a comprehensive set of fused features. These fused features were input into a self-attention module to adjust their weights according to the prediction tasks. Finally, average pooling was applied to yield patient-level features for downstream prediction tasks.

### 2.7 Prediction task for PD-L1 level

Given the fused multimodal features, a network with two fully-connected layers was used to predict PD-L1 levels. Based on the PD-L1 levels measured by immunohisto-chemistry, we divided the 220 patients into two groups: a high-level group (PD-L1≥50, *n*=32) and a low-level group (PD-L1*<*50, *n*=188). The patients in the high-level group were labeled as 1, and 0 otherwise. As a result, we formulated the prediction task as a binary classification problem. The cross-entropy loss function was used:


$$ℒ=-\;\sum_{i}y_{i}\text{ log }p_{i}+\left(1-y_{i}\right)\;\text{log }\left(1\;-p_{i}\right)$$

where $y_{i}$ is the ground-truth label of PD-L1, $p_{i}$ is the predicted probability. In this task, we excluded the clinical variables during the feature fusion stage, as clinical information contributed less to predicting PD-L1 levels.

We trained our model using the Adam optimizer for a total of 50 epochs. The initial learning rate was set to 1e-5, with the learning rate multiplied by 0.1 every 15 epochs. The weight decay was set to 1e-2. We employed five-fold cross-validation to verify the performance of our model and reported the average AUC value.

### 2.8 Prediction task for overall survival

For overall survival (OS) prediction, the patients in the PD-L1 cohort were divided into two groups based on their OS using a threshold of 12 months or 36 months. With the 12-month threshold, patients were categorized into a high-risk group (OS<12 months, *n*=24) and a low-risk group (OS≥12 months, *n*=196). Using the 36-month threshold, patients were also categorized into a high-risk group (OS<36 months, *n*=67) and a low-risk group (OS≥36 months, *n*=153). The patients in the high-risk group were labeled as 1, and 0 otherwise. Similarly, the binary cross-entropy loss function was used for the OS prediction task.

### 2.9 Prediction task for immunotherapy response

For the 75 patients in the immunotherapy cohort, we categorized patients with partial response (PR) and complete response (CR) as responders (*n*=36, labeled as 1), and those with stable disease (SD) as non-responders (*n*=39, labeled as 0). We were also interested in the prediction of progression-free survival (PFS). For this task, the patients with PFS less than 12 months were classified into a low-PFS group (*n*=27, PFS<12 months), and others were classified into a high-PFS group (*n*=48, PFS≥12 months). The prediction task was formulated as a binary classification task, utilizing the cross-entropy loss function.

Since the expression level of PD-L1 is closely related to immunotherapy response (28, 29), with high PD-L1 levels generally indicating a favorable response to immunotherapy, we incorporated the features derived from the PD-L1 level prediction model into the immunotherapy response prediction task. Specifically, the 512-dimensional features, transferred from the final layer of the trained PD-L1 prediction model, were concatenated with 8-dimensional radiomic features and 8-dimensional clinical variables selected by the Lasso algorithm. The combined features were fed into a single fully-connected layer to predict immunotherapy response.

### 2.10 Integration of longitudinal CT images

To further enhance the predictive performance for immunotherapy response, we introduced a recurrent neural network (RNN) module (30) into the multimodal framework to effectively exploit the longitudinal CT images. First, we used the Pyradiomics package to extract radiomic features from the baseline and the early on-treatment CT images. These radiomic features were then converted into 64-dimensional embeddings through a fully-connected network. The embeddings were stacked over time to form a sequence of input features for the RNN module. Finally, we concatenated the pathological features, longitudinal CT features, and clinical variables into a 128-dimensional fused feature, which was subsequently used to predict the immunotherapy response.

### 2.11 Feature importance assessment

We used the Shapley Additive Explanations (SHAP) method to evaluate the importance of features (31–33). The SHAP value measures the impact of each feature on the predictions of a machine learning model for a single input. The average SHAP value across a dataset quantifies the overall importance of an input feature. The Kernel Explainer function of SHAP was used to assess the importance of clinical variables and deep learning-derived features. Bar plots were utilized to depict the average SHAP value magnitudes of top variables for each class. For each variable group, total importance is defined as the sum of the importance across all variables in that group (e.g., all clinical variables or deep learning features). To enhance interpretability for deep learning features, the class activation map (CAM) (34) was employed to visualize the most important features.

## 3 Results

### 3.1 Multimodal fusion enhanced PD-L1 level prediction

**Figure 2:**
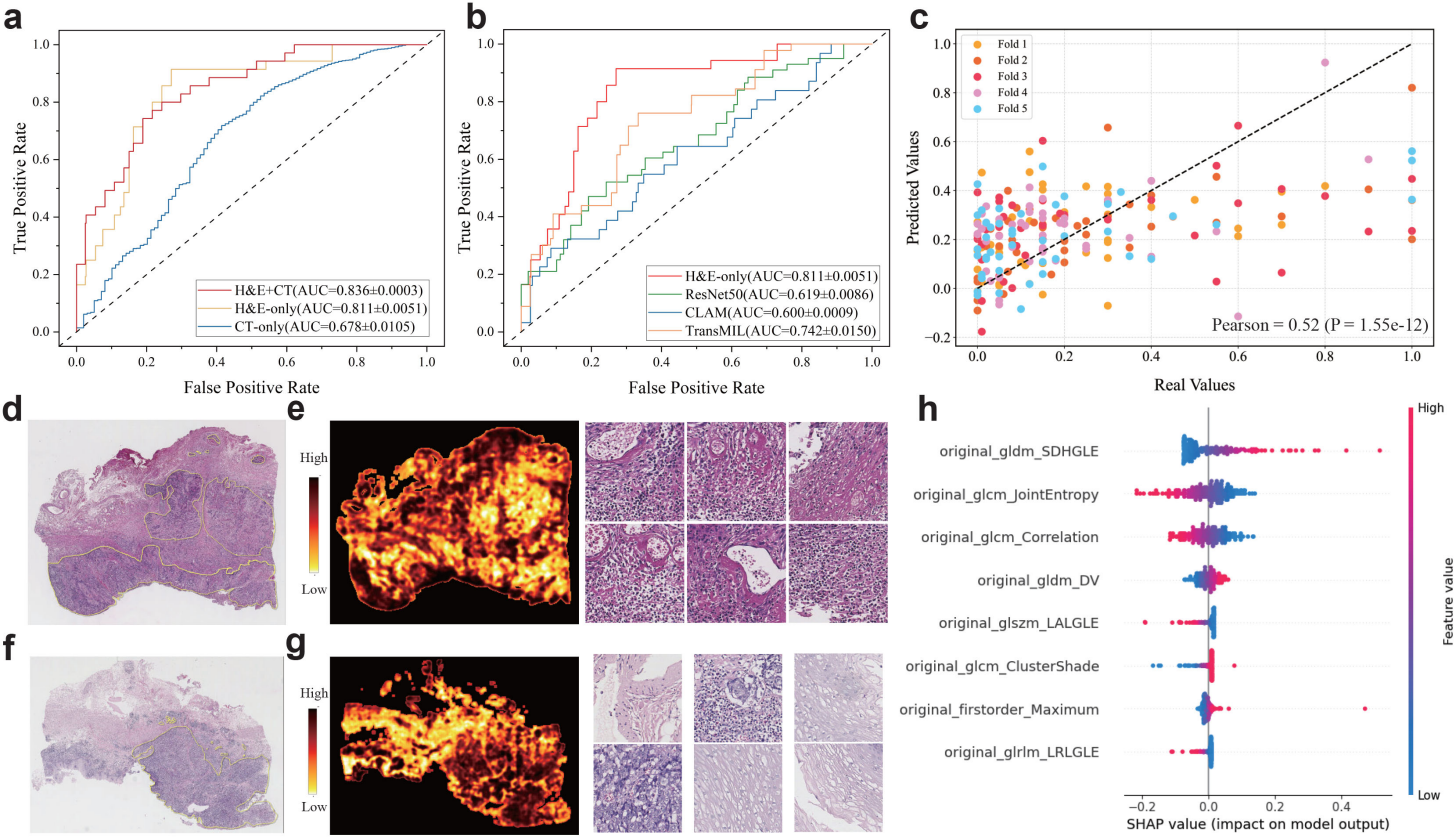
Multimodal prediction of PD-L1 levels in ESCC cohort. **(a, b)** ROC curves for the prediction of PD-L1 level (high: CPS≥50 vs low: CPS<50) using the multimodal and ablated models. **(b)** ROC curves for performance comparison between our image-only model and comparative methods. **(c)** Scatter plots of the predicted and actual expression levels of PD-L1. **(d-g)** Representative H&E slides associated to the specimens with low and high PD-L1 levels assessed by immunohistochemistry (upper: low PD-L1 and bottom: high PD-L1), as well as the heatmaps generated by predicted scores and exemplar patches. **(h)** SHAP values of top predictive features for PD-L1 levels. The features are ranked by the sum of SHAP value magnitudes over all samples.

For PD-L1 level prediction, our multimodal model achieves superior performance in distinguishing high-level and low-level cases at the threshold 50 CPS (AUC=0.836 ± 0.0003, **Figure 2a**). To further demonstrate the effectiveness of the multimodal model, we built a few variant models that used only H&E images or CT images. The results showed that the multimodal model surpassed the H&E-only model (AUC=0.81 ± 0.0051) and the CT-only model (AUC=0.678 ± 0.0105). Also, we compared our H&E-only model to previously published methods that are also built solely on H&E slides (**Figure 2b**), including ResNet50 (35), CLAM (36), and TransMIL (37). We found that our H&E-only model remarkably outperformed ResNet50 (AUC=0.66 ± 0.0086), CLAM (AUC=0.61 ± 0.0009), and TransMIL (AUC=0.74 ± 0.0150).

Furthermore, we used the multimodal model to perform regression analysis on PD-L1 levels. For this regression task, the PD-L1 levels were normalized into the [0, 1] range, and the Pearson correlation coefficient (*r*) (38) was reported as a performance metric. As a result, our multimodal model showed notable performance (**Figure 2c**), and we observed a significant positive correlation between the actual and predicted values across five-fold cross-validation (*r*=0.52, *p*-value=1.55e-12).

To explore the impact of patch-level features on the PD-L1 prediction, we evaluated the importance of each patch by calculating the ratio of the predicted probabilities with and without the inclusion of each patch’s feature. This ratio reflected the importance of each patch and enabled us to generate a heatmap for each slide. We presented two representative H&E slides randomly selected from the PD-L1 cohort, along with the corresponding heatmaps and several patches with high and low importance scores (**Figure 2d–g**). Visual inspection of these patches revealed significant differences, suggesting close association between the PD-L1 levels and histological morphology. Furthermore, we highlighted the important radiomic features evaluated by SHapley Additive exPlanation (SHAP) (31–33) values (**Figure 2h**) that contribute significantly to PD-L1 prediction.

### 3.2 Multimodal prediction of overall survival

**Figure 3:**
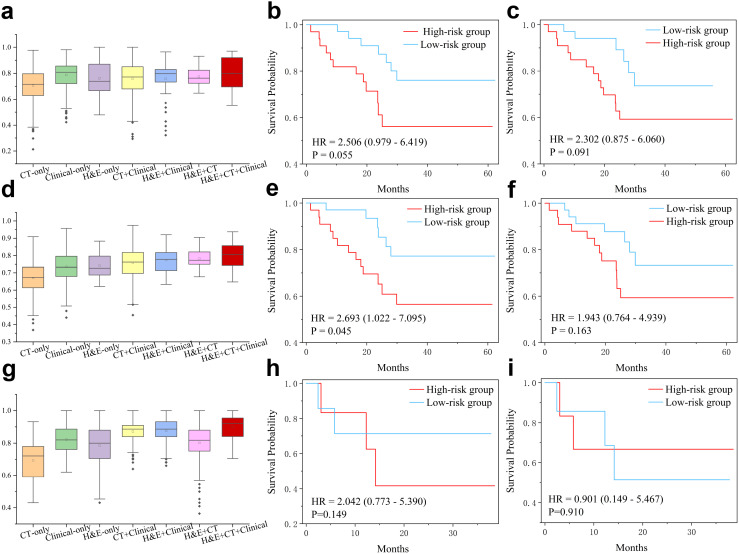
Multimodal features for prediction of overall survival (OS) and progression-free survival (PFS). **(a)** AUC values in prediction of high-risk (OS<12 months) and low-risk (OS≥12 months) patients of the PD-L1 cohort. Note that statistical significance has been conduced by two-sided *t-*test and the results are presented in the **Supplementary Table S1**. **(b, c)** Kaplan-Meier curves for hig-hand low-risk (cutoff=12m) patients predicted by the multimodal and H&E-only models, respectively. **(d)** AUC values in prediction of high-risk (OS<36 months) and low-risk (OS≥36 months) patients of the PD-L1 cohort. Statistical significance (two-sided *t*-test) has been presented in the **Supplementary Table S2**. **(e, f)** Kaplan-Meier curves for high- and low-risk (cutoff=36m) patients predicted by the multimodal and H&E-only models, respectively. **(g)** AUC values in prediction of high- and low-PFS (cutoff=12m) patients of immunotherapy cohort. Statistical significance (two-sided *t*-test) has been presented in the **Supplementary Table S3**. **(h, i)** Kaplan-Meier curves for high- and low-PFS (cutoff=12m) patients of immunotherapy cohort, predicted by the multimodal model and pre-trained model on PD-L1 cohort, respectively.

We further evaluated the multimodal model in predicting overall survival. For the high-and low-risk groups defined by a 1-year threshold, we compared the multimodal model with variant models built on different subsets of features (**Figure 3a**). Although the model using only clinical variables (AUC=0.785 ± 0.0103) performed better than the H&E-only model (AUC=0.761 ± 0.014) or the CT-only model (AUC=0.705 ± 0.015), the multimodal fusion achieved the highest performance (AUC=0.802 ± 0.014), which also outperformed the clinical+CT model (AUC=0.756 ± 0.015), the clinical+H&E model (AUC=0.751 ± 0.023), and the H&E+CT model (AUC=0.766 ± 0.005). Furthermore, based on the multimodal model predicted scores, we categorized the patients into low-risk and high-risk groups and conducted survival analysis (**Figure 3b**, **Supplementary Figure S1**). The Kaplan–Meier curves showed that the high-risk group had a poorer prognosis compared to the low-risk group (HR=2.10, *p*-value=0.055), where HR represents the hazard ratio between the high-risk and low-risk groups, indicating the relative risk of an event occurring in the high-risk group. However, the stratification by H&E-only model and other ablated models did not show statistical significance (**Figure 3c**, **Supplementary Figure S1**).

Using the 3-year threshold, the H&E-only model (AUC=0.741 ± 0.004) outperformed the model using only clinical variables (AUC=0.735 ± 0.008) or the model using radiomics features (AUC=0.670 ± 0.008) (**Figure 3d**). The multimodal model demonstrated superior performance (AUC=0.802 ± 0.004), compared to the clinical+CT model (AUC=0.758 ± 0.007), H&E+clinical model (AUC=0.774 ± 0.097), and H&E+CT model (AUC=0.782 ± 0.098). Similarly, the survival analysis based on the predicted scores by the multimodal model showed that the high-risk group had a poorer prognosis than the low-risk group (HR=2.460, *p*-value=0.045), while all other ablated models did not yield statistical significance (**Figures 3e, f**, **Supplementary Figure S2**).

Furthermore, the multimodal model also demonstrated superior performance in the PFS prediction task (**Figures 3g–i**). The patients were classified into high- and low-PFS groups using a threshold of 12 months. As a result, the clinical-only model achieved better performance (AUC=0.820 ± 0.004) than the H&E-only model (AUC=0.713 ± 0.021) and the CT-only model (AUC=0.698 ± 0.028). The multimodal model showed the best performance (AUC=0.875 ± 0.010), which is better than the clinical+CT model (AUC=0.857 ± 0.009), H&E+clinical model (AUC=0.856 ± 0.006), and H&E+CT model (AUC=0.757 ± 0.027).

**Figure 4:**
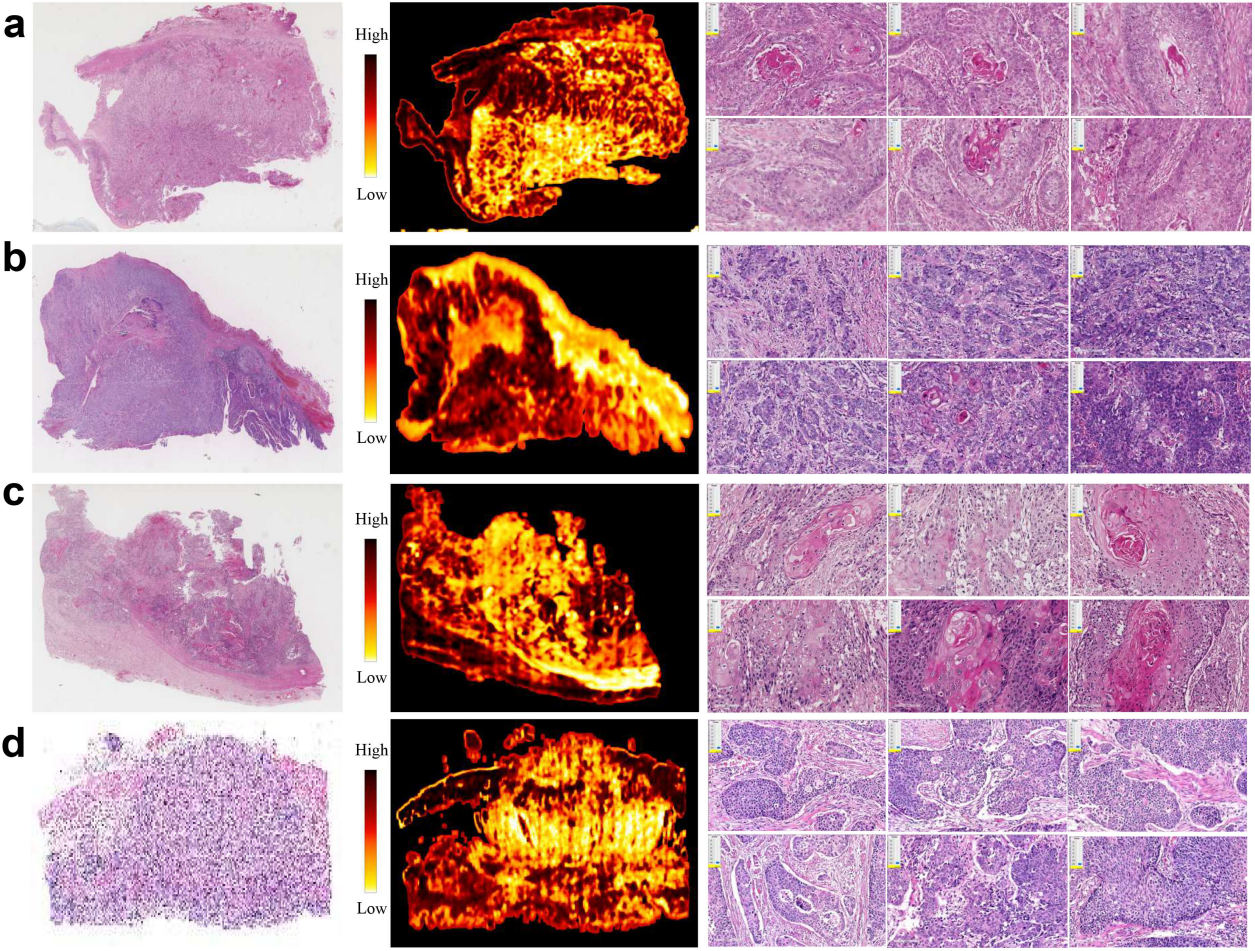
Representative H&E slides from PD-L1 cohort, and corresponding heatmaps generated using patch-level attention scores, as well as exemplar patches. **(a, b)** H&E slides, heatmaps and exemplar patches randomly selected from patients with high- and low-risk prognosis by threshold 12 months, respectively. **(c, d)** H&E slides, heatmaps and exemplar patches randomly selected from patients with high- and low-risk prognosis by threshold 36 months, respectively.

To reveal key features with prognostic values, we computed SHAP values of multi-modal features in prediction of OS (**Supplementary Figure S3**). Some radiomic features associated with tumor shape and first-order statistics contributed significantly to OS. Quite a few clinical variables, such as hypertension, hemoglobin, and stage, played a key role in OS prediction. Moreover, we presented four representative H&E slides, which were randomly selected from the low-risk and high-risk patients for 1-year OS and 3-year OS. For visual inspection, we generated their corresponding heatmaps and presented a few representative patches for each slide (**Figures 4a–d**). After careful examination, pathologists concluded that the high-risk slides were characterized by lack of keratin pearls, numerous mitotic figures, increased tumor cellularity and intensity. In contrast, the slides from the low-risk group exhibited formation of keratin pearls and intercellular bridges.

### 3.3 Multimodal prediction of immunotherapy response

**Figure 5:**
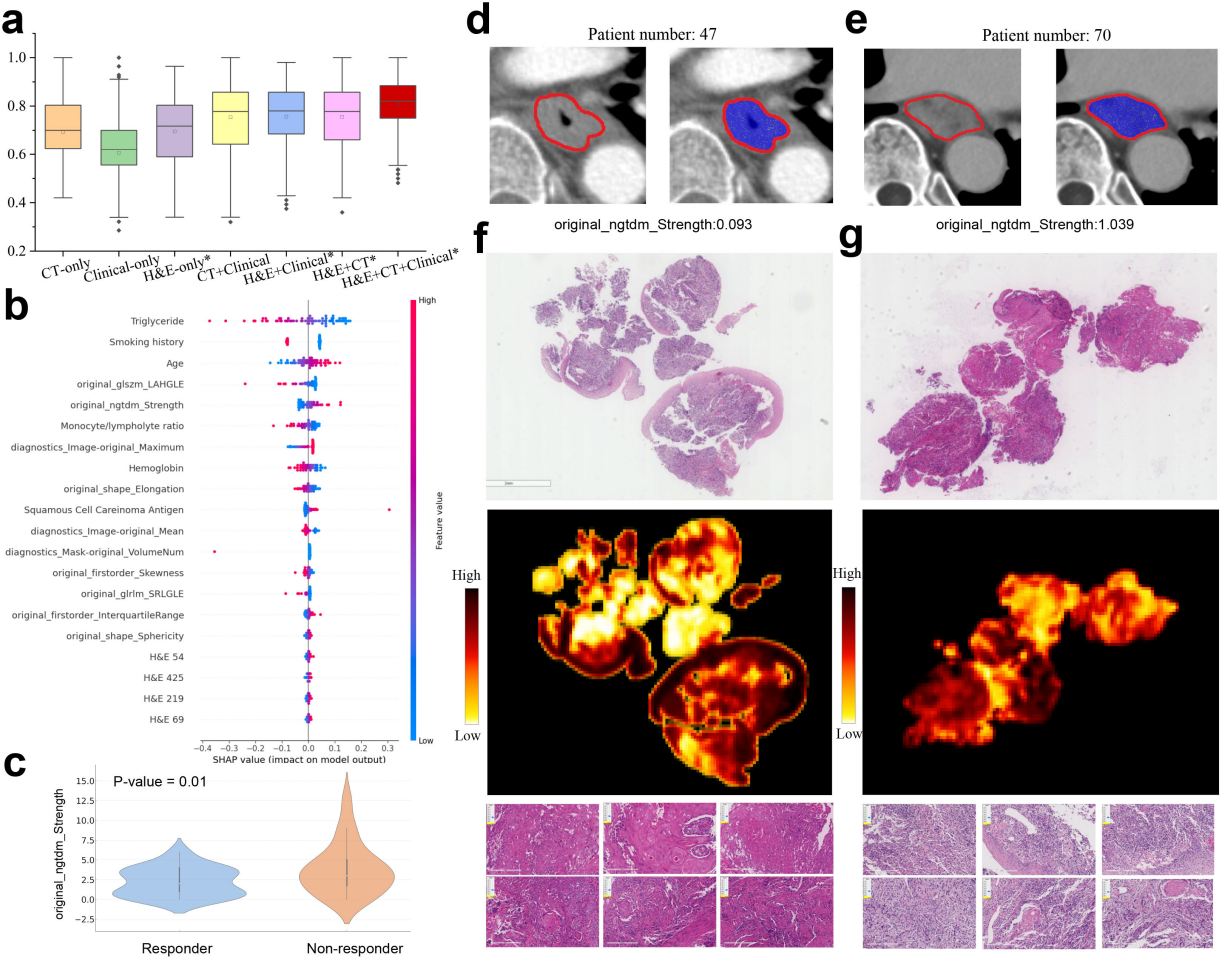
Multimodal prediction of immunotherapy response. **(a)** AUC values in prediction of responsive and non-responsive to immunotherapy by multimodal and ablated models. The models marked with an asterisk use the pathological features learned in PD-L1 prediction task. Statistically significant differences are detailed in the**Supplementary Table S4**. **(b)** Top 20 multimodal features ranked by SHAP values for predicting immunotherapy response. **(c)** Violin plot of the radiomic feature original ngtdm Strength in responders and non-responders. **(d, e)** CT images with annotated tumor regions came from two patients having low and high original ngtdm Strength feature values. **(f, g)** Representative H&E slides came from responders and non-responders to immunotherapy (left column: responsive, right column: non-responsive), as well as the corresponding heatmaps and exemplar patches.

To highlight the significance of multimodal data in predicting immune therapy response, we initially developed predictive models using basic features and assessed their performance. We then progressively fused features from diverse modalities to improve model performance. Specifically, we initially predicted immunotherapy response using only H&E and CT feature, achieving a suboptimal performance with an AUC of 0.65. After integrating the pathological features extracted from H&E for predicting PD-L1 level, the AUC improved to 0.70. When these fused features were further combined with clinical variables, the AUC increased to 0.75. Finally, the incorporation of radiomics features resulted in a further improvement, achieving an AUC of 0.80 (**Figure 5a**).

To investigate the interpretability of our model, we computed SHAP values to assess the contribution of the clinical variables, H&E features, and radiomic features to the final prediction (**Figure 5b**). Several clinical variables, such as smoking history, age, and hemoglobin (Hb) levels, significantly contributed to the prediction of immunotherapy response. Importantly, some radiomic features, originally transferred from the PD-L1 prediction task, also played a significant role in influencing the immunotherapy response. For example, the GLSZM LAHGLE feature, which quantifies the proportion of the joint distribution of larger zones with higher grey-level values within the tumor, often reflects the tumor intratumoural heterogeneity associated with the response to treatment. Moreover, the NGTDM strength feature was notably related to the treatment response. The responders exhibited significantly lower NGTDM strength values compared to non-responders (**Figure 5c**, *p*-value=0.01). Further analysis of two representative cases (**Figures 5d, e**) revealed that the case with high NGTDM strength (right, non-responder) showed well-defined boundaries and a uniform internal structure, in contrast to the case with low NGTDM strength (left, responder). Examination of corresponding H&E slides (**Figures 5f, g**) indicated that the non-responder slide was characterized by a lack of keratin pearl formation, high mitotic figures, increased tumor cellularity, and intensity. In addition, the clinical variables such as smoking history, triglyceride, hemoglobin, and age also contributed significantly to the immunotherapy response prediction.

### 3.4 Early on-treatment CT enhanced prediction performance

Although the multimodal model achieved an AUC of 0.8 in the prediction of immunotherapy response, there remains a significant gap for clinical practice. We further tested whether the first follow-up CT after treatment could enhance predictive performance. Our findings indicate that by integrating longitudinal CT features with H&E and clinical features, the multimodal model achieved an AUC of 0.937 ± 0.002 (**Supplementary Figure S4A**). Treatment with immune checkpoint inhibitors typically lasted between 2 and 6 months, with an average duration of 4 months. The first follow-up CT is usually conducted around 1-2 months after the start of treatment. Our study verified that the incorporation of the early on-treatment CT scans remarkably improved the predictive performance, suggesting that longitudinal CT effectively captured the changes in characteristics of lesions induced by immunotherapy.

To explore the important features, we employed the SHAP method to assess the contribution of each clinical and deep learning feature to the model prediction (**Supplementary Figure S4B**). It was found that the radiomic features extracted from longitudinal CT scans accounted for the top 20 most important features. In contrast, clinical variables such as smoking history, triglyceride, hemoglobin, and age contributed less significantly to the immunotherapy response prediction.

## 4 Discussion

The PD-L1 level has gained attention as a predictive biomarker for immunotherapy response. Previous deep learning-based studies focus on predicting PD-L1 biomarker from H&E-stained slides across several cancer types. For instance, one study achieved a weighted average AUC of 0.74 on formalin-fixed specimens across nine types of tumors where PD-L1 is an established biomarker (14). However, the use of CT images for PDL1 level prediction has been less explored. A pioneering study proposed a deep learning model to predict PD-L1 expression using CT images in non-small cell lung cancer (NSCLC) patients, achieving AUCs of 0.71 (95% CI: 0.59-0.81) and 0.76 (95% CI: 0.66-0.85) in the validation and test cohorts (4). Despite these efforts, the performance of such studies has not been satisfactory. In contrast, our multimodal deep learning model achieved 0.836 AUC value in predicting PD-L1 levels of ESCC patients. Our study demonstrated that H&E staining and CT imaging are highly indicative of PD-L1 expression, and these predictive signatures can be effectively learned by an adequately trained deep learning model based on unannotated samples. From a clinical practice perspective, our multimodal model offers a cost-effective and efficient alternative to traditional immunohistochemistry (IHC) techniques.

Our model successfully stratified patients who underwent surgery into low- and high-risk groups in terms of overall survival. For the prediction of 1-year and 3-year survival times, our model achieved AUC values exceeding 0.80, underscoring the robustness of the proposed model. Compared to previous studies that relied primarily on single-modal data, such as the H&E slides (39, 40) or CT images (41), our multimodal model demonstrated superior performance. Previous studies have utilized tumor characteristics (e.g., location, size, differentiation, TNM stage) and pathology features (e.g., lymphovascular invasion), as well as hematology test results (e.g., leukocyte and platelet counts) to predict clinical outcomes of ESCC patients (41). However, CT images or clinical variables alone are insufficient to fully reflect the complexity of clinical outcomes. In contrast, our multimodal model, which integrated diverse data sources, provided complementary information that enhanced predictive capability in a clinical setting.

We also evaluated our multimodal model for predicting immunotherapy response on a separate ESCC immunotherapy cohort, entirely independent from the PD-L1 cohort. Notably, we found that the multimodal features extracted by the encoder, trained on the PD-L1 cohort for PD-L1 level prediction, significantly enhanced the predictive performance for immunotherapy response. This finding aligned with previous reports indicating that higher PD-L1 levels are often associated with a more favorable response to immunotherapy (5). The cross-cohort experiments confirmed that our model captures features pertinent to the prediction tasks, rather than merely memorizing the samples. Consequently, our multimodal model achieved an AUC value exceeding 0.8 in predicting immunotherapy response, demonstrating its efficacy in stratifying patients likely to benefit from immunotherapy.

One limitation of this study is that the current model is restricted to binary classification, whereas immunotherapy response is typically categorized into four distinct types: complete response, partial response, stable disease, and progressive disease. Furthermore, the sample size of ESCC patients in the cohorts is relatively small, which may affect the robustness and generalizability of the model. In addition, incorporating multi-omics data, such as genetic alterations and epigenetic modifications, could potentially enhance the model’s performance in predicting clinical outcomes.

## 5 Conclusion

We propose a multimodal deep learning model designed to predict PD-L1 biomarker level and immunotherapy response in patients with esophageal squamous cell carcinoma. Our approach integrates multimodal features derived from Hematoxylin and Eosin (H&E) stained slides and CT images, alongside clinical variables. The integrated features are highly indicative of PD-L1 expression levels, immunotherapy response, and overall survival. Furthermore, our findings reveal that extracted features predictive of PD-L1 expression are also significantly associated with immunotherapy response. Notably, the inclusion of longitudinal CT images enhances the predictive accuracy of immunotherapy response.

## Data Availability

The datasets presented in this study can be found in online repositories. The names of the repository/repositories and accession number(s) can be found in the article/**Supplementary Material**.
