# Supplementary material for: Multimodal deep learning for predicting PD-L1 biomarker and clinical immunotherapy outcomes of esophageal cancer
Source: Front Immunol. 2025 Mar 11;16:1540013. doi: 10.3389/fimmu.2025.1540013 (PMC11933072; doi:10.3389/fimmu.2025.1540013)
Supplement: Supplementary file 1 [file DataSheet1.docx]

The source codes used in this study are available at: https://github.com/hliulab/multimodal.

Data:https://www.jianguoyun.com/p/DQ3m0XkQ8vHqCRiPwO4FIAA
