# Supplementary material for: Multimodal deep learning for predicting PD-L1 biomarker and clinical immunotherapy outcomes of esophageal cancer
Source: Front Immunol. 2025 Mar 11;16:1540013. doi: 10.3389/fimmu.2025.1540013 (PMC11933072; doi:10.3389/fimmu.2025.1540013)
Supplement: Supplementary file 2 [file DataSheet2.pdf]

Supplementary file

February 18, 2025

1 Supplementary Tables

Table S1: Statistically significant differences (p-values) between multimodal and ablated models in the 12-month OS prediction task(p-values were calculated using two-tailed t-tests to compare the performance of different models)

	Cli	H&E	CT+Cli	H&E+Cli	H&E+CT	H&E+Cli+CT
CT	**4.41e-28**	**4.91e-13**	**6.22e-11**	**2.25e-04**	**1.07e-07**	**4.71e-34**
Cli		**4.77e-04**	**4.82e-05**	**1.95e-02**	2.34e-01	**2.20e-02**
H&E			5.48e-01	7.69e-01	3.34e-01	**9.26e-08**
CT&Cli				9.69e-01	1.94e-01	**5.28e-09**
H&E+Cli					3.40e-01	**1.22e-03**
H&E+CT						**2.03e-02**

Table S2: Statistically significant differences (p-values) between multimodal and ablated models in 36-month OS prediction task(p-values were calculated using two-tailed t-tests to compare the performance of different models)

	Cli	H&E	CT+Cli	H&E+Cli	H&E+CT	H&E+Cli+CT
CT	**4.38e-29**	**1.22e-43**	**4.30e-51**	**1.49e-24**	**7.97e-30**	**2.13e-116**
Cli		1.80e-01	**2.50e-05**	**3.39e-05**	**1.93e-07**	**1.00e-39**
H&E			**4.04e-04**	**3.91e-06**	**5.89e-10**	**8.69e-47**
CT+Cli				7.14e-02	**4.64e-03**	**4.26e-20**
H&E+Cli					3.56e-01	**1.42e-04**
H&E+CT						**3.61e-03**

Table S3: Statistically significant differences (p-values) between multimodal and ablated models in the 12-month PFS prediction task(p-values were calculated using two-tailed t-tests to compare the performance of different models)

	Cli	H&E	CT+Cli	H&E+Cli	H&E+CT	H&E+Cli+CT
CT	**3.30e-82**	**6.85e-35**	**4.63e-142**	**6.56e-139**	**1.05e-46**	**2.52e-162**
Cli		**5.93e-10**	**2.22e-27**	**9.06e-28**	**9.00e-32**	**3.38e-51**
H&E			**3.16e-64**	**3.75e-46**	1.94e-1	**7.04e-46**
CT+Cli				**3.05e-67**	**9.00e-32**	**2.98e-09**
H&E+Cli					**1.75e-32**	**1.03e-06**
H&E+CT						**1.08e-51**

Table S4: Statistically significant differences (p-values) between multimodal and ablated models for immunotherapy response prediction using pre-treatment CT scans(p-values were calculated using two-tailed t-tests to compare the performance of different models)

	Cli	H&E	CT+Cli	H&E+Cli	H&E+CT	H&E+Cli+CT
CT	**3.78e-23**	8.71e-01	**5.09e-12**	**3.69e-14**	**3.70e-13**	**8.19e-48**
Cli		**5.59e-23**	**2.99e-54**	**5.91e-61**	**1.53e-57**	**1.19e-111**
H&E			**3.58e-11**	**4.10e-13**	**3.24e-12**	**8.99e-45**
CT+Cli				7.89e-01	8.49e-01	**5.47e-12**
H&E+Cli					9.42e-01	**1.43e-12**
H&E+CT						**5.60e-12**

Table S5: Statistically significant differences (p-values) between multimodal and ablated models for immunotherapy response prediction using early on-treatment CT scans(p-values were calculated using two-tailed t-tests to compare the performance of different models)

	Longitudinal+Cli	Longitudinal+H&E	Longitudinal+H&E+Cli
Longitudinal	**5.36e-08**	**1.43e-12**	**5.29e-10**
Longitudinal+Cli		**1.11e-02**	1.92e-01
Longitudinal+H&E			2.46e-01

Table S6: Clinical and radiomics data collected from the ESCC PD-L1 cohort and the ESCC Immunotherapy cohort

		ESCC PD-L1 cohort		ESCC Immunotherapy cohort	
		PD-L1<50% (N=188)	PD-L1≥ 50%(N = 32)	Non-responder (N=39)	Responder (N=36)
OS(months)		24.43	26.03		
PFS(months)				13.33	14.42
Age		66.7 ± 8.1	65.4 ± 7.0	68.23 ± 8.73	65.75 ± 8.31
Gender	Female	37(20)	8(25)	4(10)	6(17)
	Male	151(80)	24(75)	35(90)	30(83)
BMI		22.53 ± 3.28	22.76 ± 3.51	21.93 ± 2.97	22.02 ± 2.86
Smoking history	No	109(58)	18(56)	28(72)	21(58.33)
	Yes	79(42)	14(44)	11(28)	15(41.67)
Drinking history	No	115(61)	21(66)	30(76)	25(70)
	Yes	73(39)	11(34)	9(23)	11(30)
Hypertension	No	106(56)	18(56)	27(70)	22(61)
	Yes	82(44)	14(44)	12(30)	14(39)
Adjuvant radiotherapy	No	117(62)	18(56)		
	Yes	71(37)	14(44)		
Adjuvant chemotherapy	No	107(57)	14(44)		
	Yes	81(43)	18(56)		
Treatment strategy	Immunotherapy			4(10)	0(0)
	Immunotherapy + Chemotherapy			35(90)	36(100)
TNM Stage	I	16(9)	3(9)		
	II	83(44)	12(38)		

	III	78(41)	16(50)	25(64)	24(66)
	IV	11(6)	1(3)	14(36)	12(33)
Grade	G1	15(8)	5(16)		
	G2	124(66)	15(47)		
	G3	49(26)	12(37)		
Neurovascular invasion	No	104(55)	19(60)		
	Yes	84(45)	13(40)		
Tumor Size		12.21 ± 9.76	12.89 ± 7.74		
PD-L1/PD-1 inhibitor	Sintilimab			30(77)	29(80)
	Camrelizumab			4(10)	6(17)
	Tislelizumab			5(13)	1(3)
WBC		6.19 ± 1.77	6.21 ± 2.62	6.06 ± 1.63	5.95 ± 1.47
PLT		220.18 ± 70.35	206.26 ± 72.91	219.83 ± 63.22	219.36 ± 83.11
Hb		134.50 ± 13.99	129.81 ± 18.92	131.14 ± 18.65	126.50 ± 15.13
SII		598.82 ± 417.47	603.64 ± 575.70	695.30 ± 406.41	756.74 ± 643.61
PLR		146.26 ± 62.51	142.00 ± 48.78	165.20 ± 72.43	193.33 ± 129.21
NLR		2.63 ± 1.23	2.93 ± 2.02		
MLR		0.27 ± 0.10	0.29 ± 0.12	0.32 ± 0.14	0.35 ± 0.20
NEUT		3.92 ± 1.44	4.08 ± 2.39	4.14 ± 1.40	3.76 ± 1.26
LYM		1.64 ± 0.57	1.57 ± 0.67	1.45 ± 0.50	1.37 ± 0.53
MONO		0.42 ± 0.16	0.41 ± 0.17	0.44 ± 0.17	0.41 ± 0.16
EOS		0.18 ± 0.22	0.16 ± 0.17	0.16 ± 0.17	0.15 ± 0.14
BASO		0.03 ± 0.02	0.03 ± 0.02	0.03 ± 0.02	0.03 ± 0.02
NEUT%		62.44 ± 9.14	63.27 ± 10.28	64.50 ± 9.97	67.30 ± 9.25
EOS%		2.93 ± 2.52	2.67 ± 3.04	2.62 ± 2.76	2.56 ± 2.48
BASO%		0.53 ± 0.26	0.54 ± 0.26	0.55 ± 0.38	0.48 ± 0.27
LYM%		27.39 ± 8.61	26.67 ± 9.39	25.15 ± 8.41	22.38 ± 8.21
Scr		71.34 ± 12.08	74.84 ± 21.58	78.92 ± 25.72	70.51 ± 11.90
TC		4.46 ± 0.86	4.50 ± 0.90	4.39 ± 0.90	4.49 ± 0.76
TG		1.35 ± 0.71	1.30 ± 0.62	1.15 ± 0.54	1.19 ± 0.42
HDL		1.14 ± 0.33	1.13 ± 0.25	1.19 ± 0.31	1.16 ± 0.26
LDL		2.64 ± 0.73	2.70 ± 0.77	2.64 ± 0.76	2.73 ± 0.69
ApoA1		1.17 ± 0.29	1.16 ± 0.20	1.17 ± 0.33	1.14 ± 0.22

ApoB		0.91 ± 0.25	0.94 ± 0.25	0.93 ± 0.27	0.96 ± 0.25
PA		129.32 ± 40.91	119.96 ± 47.65		
CA-199				15.31 ± 21.85	9.50 ± 7.21
CA-125				15.96 ± 17.95	9.51 ± 3.99
CEA				2.73 ± 1.68	3.56 ± 7.90
SCCAg				2.95 ± 2.51	4.34 ± 11.33
diagnostics Image-original Mean		-814.08 ± 222.19	-806.55 ± 221.74	-775.36 ± 200.73	-819.80 ± 217.34
diagnostics Image-original Minimum		-1675.64 ± 943.48	-1618.50 ± 911.20	-1441.33 ± 806.73	-1588.10 ± 911.76
diagnostics Image-original Maximum		2716.60 ± 1001.32	2824.47 ± 593.91	2629.67 ± 624.24	2861.69 ± 450.08
diagnostics Mask-original VoxelNum		5475.43 ± 3959.31	5798.56 ± 4140.11	8710.61 ± 6273.11	7572.72 ± 7545.26
diagnostics Mask-original VolumeNum 39(100)		1.00 ± 0.00	1.00 ± 0.00	35(97)	1(3)
original shape Elongation		0.54 ± 0.15	0.55 ± 0.13	0.54 ± 0.13	0.48 ± 0.15
original shape Flatness		0.38 ± 0.10	0.38 ± 0.11	0.39 ± 0.11	0.34 ± 0.09
original shape LeastAxisLength		17.87 ± 4.46	17.93 ± 4.28	21.36 ± 5.96	19.65 ± 4.96
original shape MajorAxisLength		50.78 ± 17.53	50.32 ± 15.90	57.92 ± 17.54	61.33 ± 18.94
original shape Maximum2DDiameterColumn		50.48 ± 15.89	51.84 ± 16.50	59.39 ± 16.76	58.81 ± 18.62
original shape Maximum2DDiameterRow		51.96 ± 17.44	51.63 ± 16.26	58.88 ± 17.07	62.29 ± 18.59
original shape Maximum2DDiameterSlice		30.28 ± 8.03	31.72 ± 8.34	35.32 ± 8.58	32.98 ± 8.55
original shape Maximum3DDiameter		55.38 ± 17.18	56.00 ± 16.61	62.97 ± 16.66	66.03 ± 18.90
original shape MeshVolume		17779.91 ± 13293.99	18233.23 ± 12515.92	28371.13 ± 21672.88	24920.02 ± 20112.49
original shape MinorAxisLength		25.58 ± 6.96	26.62 ± 7.50	29.75 ± 7.28	27.85 ± 7.17
original shape Sphericity		0.70 ± 0.05	0.70 ± 0.05	0.71 ± 0.05	0.69 ± 0.05
original shape SurfaceArea		4468.08 ± 2301.17	4586.15 ± 2195.81	5988.41 ± 3002.13	5744.77 ± 2824.39
original shape SurfaceVolumeRatio		0.30 ± 0.09	0.30 ± 0.09	0.25 ± 0.06	0.27 ± 0.06
original shape VoxelVolume		17882.82 ± 13322.89	18335.34 ± 12537.37	28490.00 ± 21699.26	25042.96 ± 20136.43
original firstorder 10Percentile		23.01 ± 42.70	33.25 ± 22.33	30.08 ± 21.65	24.84 ± 22.56

original firstorder 90Percentile		84.25 ± 17.69	84.52 ± 17.92	81.26 ± 18.60	83.70 ± 13.86
original firstorder Energy		39187912.94 ± 39674200.21	44295335.38 ± 60268788.14	68195272.14 ± 83790110.73	55725816.59 ± 72524457.45
original firstorder Entropy		2.00 ± 0.41	1.88 ± 0.37	1.91 ± 0.33	2.05 ± 0.34
original firstorder InterquartileRange		26.20 ± 8.04	23.09 ± 7.97	23.42 ± 4.83	26.35 ± 6.97
original firstorder Kurtosis		41.15 ± 30.65	51.91 ± 32.83	43.87 ± 35.03	50.63 ± 33.05
original firstorder Maximum		127.72 ± 30.83	170.56 ± 240.86	154.78 ± 161.69	134.03 ± 35.48
original firstorder MeanAbsoluteDeviation		26.91 ± 18.64	23.70 ± 12.18	23.72 ± 11.99	27.20 ± 13.22
original firstorder Mean		52.41 ± 21.17	56.88 ± 19.28	52.66 ± 20.57	51.13 ± 16.55
original firstorder Median		62.55 ± 16.10	65.08 ± 18.96	61.00 ± 16.97	61.51 ± 12.91
original firstorder Minimum		-479.85 ± 294.85	-502.78 ± 280.72	-485.36 ± 319.95	-581.64 ± 246.34
original firstorder Range		607.57 ± 302.26	673.34 ± 395.34	640.14 ± 344.21	715.67 ± 253.64
original firstorder RobustMeanAbsoluteDeviation		11.60 ± 4.52	10.07 ± 3.46	10.17 ± 2.18	11.47 ± 3.07
original firstorder RootMeanSquared		80.85 ± 27.69	80.74 ± 23.99	78.08 ± 22.17	79.93 ± 20.72
original firstorder Skewness		-4.54 ± 2.39	-4.95 ± 3.16	-4.21 ± 3.41	-5.40 ± 2.28
original firstorder TotalEnergy		126532698.06 ± 126653661.01	139852351.82 ± 182376680.03	223937542.32 ± 279712572.63	177948006.71 ± 195245428.90
original firstorder Uniformity		0.35 ± 0.08	0.38 ± 0.07	0.37 ± 0.07	0.34 ± 0.06
original firstorder Variance		4107.40 ± 6531.49	3481.20 ± 4347.59	3389.23 ± 4574.51	3925.61 ± 4537.01
original glcm Autocorrelation		630.18 ± 533.96	670.99 ± 545.57	659.64 ± 588.90	782.37 ± 466.05
original glcm ClusterProminence		36577.46 ± 91332.99	31878.37 ± 76159.34	36018.77 ± 93720.43	31273.91 ± 64499.51
original glcm ClusterShade		-736.68 ± 1621.43	-329.79 ± 1242.18	-657.61 ± 1462.42	-683.11 ± 1203.59
original glcm ClusterTendency		22.30 ± 37.96	17.30 ± 24.46	18.34 ± 27.42	22.14 ± 27.74
original glcm Contrast		5.34 ± 6.77	5.12 ± 6.02	3.95 ± 3.60	5.60 ± 6.10
original glcm Correlation		0.49 ± 0.13	0.45 ± 0.11	0.51 ± 0.12	0.54 ± 0.09
original glcm DifferenceAverage		0.92 ± 0.49	0.84 ± 0.42	0.78 ± 0.29	0.94 ± 0.45

original glcm DifferenceEntropy		1.63 ± 0.38	1.56 ± 0.35	1.53 ± 0.30	1.67 ± 0.34
original glcm DifferenceVariance		4.18 ± 5.30	4.20 ± 5.13	3.21 ± 3.09	4.47 ± 4.72
original glcm Id		0.72 ± 0.06	0.74 ± 0.07	0.74 ± 0.05	0.73 ± 0.06
original glcm Idm		0.71 ± 0.07	0.73 ± 0.08	0.73 ± 0.05	0.71 ± 0.07
original glcm Idmn		0.99 ± 0.00	0.99 ± 0.00	0.99 ± 0.00	1.00 ± 0.00
original glcm Idn		0.96 ± 0.02	0.97 ± 0.02	0.97 ± 0.01	0.97 ± 0.01
original glcm Imc1		-0.16 ± 0.04	-0.15 ± 0.04	-0.16 ± 0.03	-0.17 ± 0.04
original glcm Imc2		0.62 ± 0.12	0.59 ± 0.09	0.62 ± 0.09	0.66 ± 0.08
original glcm InverseVariance		0.39 ± 0.04	0.38 ± 0.05	0.38 ± 0.04	0.38 ± 0.03
original glcm JointAverage		22.31 ± 11.35	23.47 ± 11.00	22.52 ± 12.38	26.29 ± 9.45
original glcm JointEnergy		0.17 ± 0.07	0.20 ± 0.07	0.18 ± 0.06	0.16 ± 0.05
original glcm JointEntropy		3.62 ± 0.75	3.38 ± 0.75	3.44 ± 0.61	3.72 ± 0.66
original glcm MCC		0.57 ± 0.12	0.57 ± 0.11	0.59 ± 0.10	0.62 ± 0.08
original glcm MaximumProbability		0.31 ± 0.10	0.36 ± 0.11	0.33 ± 0.10	0.31 ± 0.09
original glcm SumAverage		44.63 ± 22.69	46.93 ± 22.01	45.03 ± 24.76	52.59 ± 18.89
original glcm SumEntropy		2.74 ± 0.50	2.57 ± 0.43	2.64 ± 0.39	2.83 ± 0.39
original glcm SumSquares		6.91 ± 11.05	5.61 ± 7.54	5.57 ± 7.66	6.94 ± 8.32
original gldm DependenceEntropy		6.06 ± 0.33	5.94 ± 0.28	6.10 ± 0.26	6.17 ± 0.22
original gldm DependenceNonUniformity		247.36 ± 163.91	259.93 ± 179.07	372.89 ± 247.35	335.71 ± 310.44
original gldm DependenceNonUniformityNormalized		0.05 ± 0.01	0.05 ± 0.01	0.04 ± 0.00	0.05 ± 0.01
original gldm DependenceVariance		35.99 ± 8.89	39.99 ± 12.54	39.97 ± 7.86	39.34 ± 8.94
original gldm GrayLevelNonUniformity		1959.15 ± 1571.69	2240.13 ± 1813.16	3219.40 ± 2480.68	2511.39 ± 2060.24
original gldm GrayLevelVariance		6.65 ± 10.45	5.66 ± 6.97	5.51 ± 7.31	6.36 ± 7.25
original gldm HighGrayLevelEmphasis		631.98 ± 536.56	671.96 ± 548.23	660.51 ± 590.67	784.58 ± 469.51
original gldm LargeDependenceEmphasis		181.27 ± 53.62	198.96 ± 64.38	209.32 ± 50.42	194.71 ± 47.66
original gldm LargeDependenceHighGrayLevelEmphasis		118033.37 ± 111253.65	131007.29 ± 109902.87	145254.36 ± 142862.29	152604.65 ± 96395.55

original gldm LargeDependenceLowGrayLevelEmphasis	1.53 ± 3.17	0.84 ± 1.18	2.12 ± 5.27	0.60 ± 0.87
original gldm LowGrayLevelEmphasis	0.01 ± 0.02	0.01 ± 0.01	0.01 ± 0.02	0.00 ± 0.00
original gldm SmallDependenceEmphasis	0.05 ± 0.03	0.05 ± 0.03	0.04 ± 0.02	0.05 ± 0.03
original gldm SmallDependenceHighGrayLevelEmphasis	29.39 ± 32.83	36.61 ± 52.62	25.24 ± 24.14	38.65 ± 51.48
original gldm SmallDependenceLowGrayLevelEmphasis	0.00 ± 0.00	0.00 ± 0.00	0.00 ± 0.00	0.00 ± 0.00
original glrlm GrayLevelNonUniformity	847.40 ± 574.07	899.79 ± 605.98	1276.94 ± 817.70	1061.86 ± 785.44
original glrlm GrayLevelNonUniformityNormalized	0.28 ± 0.06	0.29 ± 0.05	0.29 ± 0.05	0.27 ± 0.04
original glrlm GrayLevelVariance	10.06 ± 14.61	9.31 ± 11.96	9.20 ± 11.97	9.98 ± 10.96
original glrlm HighGrayLevelRunEmphasis	616.54 ± 520.98	659.62 ± 538.40	644.95 ± 574.72	766.38 ± 458.73
original glrlm LongRunEmphasis	6.10 ± 2.23	6.83 ± 2.58	7.44 ± 2.69	6.40 ± 1.94
original glrlm LongRunHighGrayLevelEmphasis	3861.13 ± 3853.70	4440.49 ± 3844.12	4992.75 ± 5284.73	4836.32 ± 3068.90
original glrlm LongRunLowGrayLevelEmphasis	0.06 ± 0.12	0.03 ± 0.05	0.09 ± 0.25	0.02 ± 0.03
original glrlm LowGrayLevelRunEmphasis	0.01 ± 0.02	0.01 ± 0.01	0.01 ± 0.02	0.00 ± 0.00
original glrlm RunEntropy	3.87 ± 0.34	3.83 ± 0.34	3.97 ± 0.29	4.01 ± 0.24
original glrlm RunLengthNonUniformity	1343.34 ± 847.89	1367.56 ± 887.87	1930.43 ± 1253.36	1864.04 ± 1884.87
original glrlm RunLengthNonUniformityNormalized	0.45 ± 0.08	0.44 ± 0.09	0.41 ± 0.06	0.43 ± 0.07
original glrlm RunPercentage	0.58 ± 0.08	0.56 ± 0.09	0.54 ± 0.07	0.56 ± 0.07
original glrlm RunVariance	2.46 ± 1.25	2.94 ± 1.50	3.23 ± 1.50	2.64 ± 1.07
original glrlm ShortRunEmphasis	0.68 ± 0.06	0.68 ± 0.07	0.66 ± 0.05	0.67 ± 0.05
original glrlm ShortRunHighGrayLevelEmphasis	418.40 ± 360.04	452.50 ± 403.98	420.90 ± 373.79	515.17 ± 335.54

original glrlm ShortRunLowGrayLevelEmphasis		0.01 ± 0.02	0.01 ± 0.01	0.01 ± 0.01	0.00 ± 0.00
original glszm GrayLevelNonUniformity		21.73 ± 11.80	24.43 ± 13.60	28.55 ± 13.55	29.28 ± 22.69
original glszm GrayLevelNonUniformityNormalized		0.13 ± 0.08	0.12 ± 0.06	0.13 ± 0.08	0.11 ± 0.05
original glszm GrayLevelVariance		34.52 ± 35.46	38.87 ± 47.70	35.66 ± 35.97	37.17 ± 26.53
original glszm HighGrayLevelZoneEmphasis		449.75 ± 364.85	528.74 ± 451.47	478.70 ± 408.31	577.76 ± 352.04
original glszm LargeAreaEmphasis		58824.25 ± 64231.42	61366.39 ± 52876.31	117282.58 ± 161504.61	70661.25 ± 67259.66
original glszm LargeAreaHighGrayLevelEmphasis		35849847.59 ± 55736813.52	43150205.94 ± 60602115.22	69820316.22 ± 111249244.13	46641822.94 ± 51814485.86
original glszm LargeAreaLowGrayLevelEmphasis		402.38 ± 854.33	178.08 ± 180.41	2765.39 ± 11902.98	311.39 ± 713.88
original glszm LowGrayLevelZoneEmphasis		0.03 ± 0.05	0.02 ± 0.03	0.03 ± 0.04	0.01 ± 0.01
original glszm SizeZoneNonUniformity		84.25 ± 76.11	94.49 ± 89.50	122.27 ± 123.27	134.97 ± 171.31
original glszm SizeZoneNonUniformityNormalized		0.34 ± 0.07	0.35 ± 0.07	0.34 ± 0.06	0.36 ± 0.07
original glszm SmallAreaEmphasis		0.60 ± 0.07	0.60 ± 0.07	0.60 ± 0.06	0.62 ± 0.07
original glszm SmallAreaHighGrayLevelEmphasis		267.26 ± 229.75	324.81 ± 303.80	283.11 ± 248.73	349.41 ± 235.45
original glszm SmallAreaLowGrayLevelEmphasis		0.02 ± 0.03	0.01 ± 0.02	0.02 ± 0.02	0.01 ± 0.01
original glszm ZoneEntropy		5.37 ± 0.75	5.39 ± 0.69	5.54 ± 0.77	5.59 ± 0.49
original glszm ZonePercentage		0.05 ± 0.03	0.05 ± 0.03	0.04 ± 0.02	0.05 ± 0.03
original glszm ZoneVariance		57876.15 ± 63556.44	60549.72 ± 52555.06	115638.78 ± 159061.76	69612.85 ± 66123.21
original ngtdm Busyness		1.79 ± 2.63	1.08 ± 0.85	4.43 ± 13.29	1.32 ± 1.78
original ngtdm Coarseness		0.00 ± 0.00	0.00 ± 0.00	0.00 ± 0.00	0.00 ± 0.00
original ngtdm Complexity		359.25 ± 407.30	457.34 ± 632.36	344.48 ± 339.47	433.61 ± 504.77
original ngtdm Contrast		0.01 ± 0.02	0.01 ± 0.01	0.01 ± 0.01	0.01 ± 0.01
original ngtd Strength		3.22 ± 3.59	3.46 ± 3.68	2.13 ± 1.80	3.76 ± 3.25

Table S7: Radiomics Data from an Immunotherapy Cohort Post-Treatment

	Non-responder (N=39)	Responder (N=36)
Column diagnostics Image-original Mean	-794.14±186.74	-826.01±184.93
Column diagnostics Image-original Minimum	-1447.11±830.42	-1456.43±834.68
Column diagnostics Image-original Maximum	3474.68±2870.65	2955.32±1160.74
Column diagnostics Mask-original VoxelNum	4451.03±2817.68	4623.46±2338.92
Column diagnostics Mask-original VolumeNum	1.00±0.00	1.03±0.16
Column original shape Elongation	0.34±0.14	0.41±0.16
Column original shape Flatness	0.25±0.11	0.29±0.11
Column original shape LeastAxisLength	15.73±3.34	17.09±3.61
Column original shape MajorAxisLength	74.98±32.62	67.09±26.06
Column original shape Maximum2DDiameterColumn	60.68±23.07	61.40±21.00
Column original shape Maximum2DDiameterRow	69.08±27.31	64.69±21.25
Column original shape Maximum2DDiameterSlice	25.46±5.06	30.18±10.89
Column original shape Maximum3DDiameter	71.58±26.93	69.01±22.35
Column original shape MeshVolume	14663.10±8238.46	17378.01±8402.12
Column original shape MinorAxisLength	21.60±4.28	24.37±4.85
Column original shape Sphericity	0.61±0.07	0.65±0.06
Column original shape SurfaceArea	4719.49±2154.45	4949.86±1837.83
Column original shape SurfaceVolumeRatio	0.35±0.06	0.31±0.06
Column original shape VoxelVolume	14776.28±8263.51	17500.07±8428.55
Column original firstorder 10Percentile	-120.09±242.32	-20.35±133.92
Column original firstorder 90Percentile	68.45±16.99	70.41±18.52
Column original firstorder Energy	93546508.68±123437899.33	40386979.97±67855207.77
Column original firstorder Entropy	2.73±0.70	2.23±0.51
Column original firstorder InterquartileRange	64.98±123.05	34.04±27.54
Column original firstorder Kurtosis	25.76±20.39	44.56±32.46
Column original firstorder Maximum	200.29±216.59	128.08±31.45
Column original firstorder MeanAbsoluteDeviation	72.84±74.11	36.47±39.13
Column original firstorder Mean	-1.88±71.48	30.12±38.60
Column original firstorder Median	36.22±25.93	46.16±20.96
Column original firstorder Minimum	-713.08±300.51	-598.41±283.25
Column original firstorder Range	913.37±353.32	726.49±287.39
Column original firstorder RobustMeanAbsoluteDeviation	32.72±54.63	16.51±20.39
Column original firstorder RootMeanSquared	133.36±98.42	83.28±55.84
Column original firstorder Skewness	-3.33±2.42	-4.87±2.44
Column original firstorder TotalEnergy	327006663.64±437599470.62	149155789.15±260345748.67
Column original firstorder Uniformity	0.25±0.08	0.30±0.07
Column original firstorder Variance	22239.02±33330.29	7611.75±16549.10
Column original glcm Autocorrelation	984.95±529.92	805.05±527.23
Column original glcm ClusterProminence	283961.57±465924.78	81655.20±245563.05
Column original glcm ClusterShade	-4438.12±6783.47	-1480.08±3932.64
Column original glcm ClusterTendency	135.49±210.71	42.79±105.77
Column original glcm Contrast	21.54±23.12	7.62±10.54
Column original glcm Correlation	0.59±0.13	0.53±0.10
Column original glcm DifferenceAverage	1.97±1.28	1.09±0.70
Column original glcm DifferenceEntropy	2.30±0.64	1.76±0.45
Column original glcm DifferenceVariance	15.57±15.37	5.83±7.57
Column original glcm Id	0.63±0.09	0.70±0.07
Column original glcm Idm	0.60±0.10	0.68±0.08
Column original glcm Idmn	0.99±0.01	0.99±0.00
Column original glcm Idn	0.96±0.02	0.97±0.01
Column original glcm Imc1	-0.19±0.05	-0.17±0.04
Column original glcm Imc2	0.75±0.11	0.68±0.10
Column original glcm InverseVariance	0.38±0.04	0.40±0.03
Column original glcm JointAverage	29.16±10.45	26.19±10.66
Column original glcm JointEnergy	0.10±0.05	0.14±0.05

Column original glcm JointEntropy	4.97±1.24	3.98±0.93
Column original glcm MCC	0.67±0.11	0.61±0.09
Column original glcm MaximumProbability	0.22±0.08	0.27±0.08
Column original glcm SumAverage	58.31±20.90	52.37±21.32
Column original glcm SumEntropy	3.61±0.83	2.99±0.60
Column original glcm SumSquares	39.26±58.03	12.60±28.88
Column original gldm DependenceEntropy	6.50±0.40	6.25±0.33
Column original gldm DependenceNonUniformity	234.17±130.05	217.00±99.72
Column original gldm DependenceNonUniformityNormalized	0.06±0.02	0.05±0.01
Column original gldm DependenceVariance	32.21±8.45	35.85±7.41
Column original gldm GrayLevelNonUniformity	1146.57±858.79	1457.59±943.52
Column original gldm GrayLevelVariance	35.63±53.28	12.26±26.46
Column original gldm HighGrayLevelEmphasis	1005.08±541.82	807.65±530.96
Column original gldm LargeDependenceEmphasis	126.65±50.09	166.19±49.48
Column original gldm LargeDependenceHighGrayLevelEmphasis	119793.38±68738.67	135712.54±97536.21
Column original gldm LargeDependenceLowGrayLevelEmphasis	1.05±2.11	0.69±1.26
Column original gldm LowGrayLevelEmphasis	0.01±0.01	0.01±0.01
Column original gldm SmallDependenceEmphasis	0.10±0.06	0.06±0.03
Column original gldm SmallDependenceHighGrayLevelEmphasis	90.49±69.80	45.18±43.36
Column original gldm SmallDependenceLowGrayLevelEmphasis	0.00±0.00	0.00±0.00
Column original glrlm GrayLevelNonUniformity	581.33±383.77	677.03±357.58
Column original glrlm GrayLevelNonUniformityNormalized	0.20±0.06	0.25±0.06
Column original glrlm GrayLevelVariance	41.24±53.79	16.12±28.57
Column original glrlm HighGrayLevelRunEmphasis	977.81±528.15	788.52±515.84
Column original glrlm LongRunEmphasis	3.84±1.55	5.28±1.89
Column original glrlm LongRunHighGrayLevelEmphasis	3478.19±1895.01	4235.88±3202.34
Column original glrlm LongRunLowGrayLevelEmphasis	0.03±0.05	0.02±0.04
Column original glrlm LowGrayLevelRunEmphasis	0.01±0.01	0.01±0.01
Column original glrlm RunEntropy	4.26±0.45	4.00±0.32
Column original glrlm RunLengthNonUniformity	1597.07±938.20	1316.78±616.21
Column original glrlm RunLengthNonUniformityNormalized	0.55±0.09	0.48±0.08
Column original glrlm RunPercentage	0.67±0.08	0.61±0.08
Column original glrlm RunVariance	1.32±0.77	2.05±1.01
Column original glrlm ShortRunEmphasis	0.76±0.07	0.71±0.06
Column original glrlm ShortRunHighGrayLevelEmphasis	749.50±422.80	553.95±373.99
Column original glrlm ShortRunLowGrayLevelEmphasis	0.01±0.01	0.01±0.01
Column original glszm GrayLevelNonUniformity	23.41±11.30	22.43±10.04
Column original glszm GrayLevelNonUniformityNormalized	0.08±0.05	0.11±0.05
Column original glszm GrayLevelVariance	71.91±49.90	44.27±41.09
Column original glszm HighGrayLevelZoneEmphasis	736.81±417.53	592.01±371.65

Column original glszm LargeAreaEmphasis	20491.73±36275.10	35178.66±37725.14
Column original glszm LargeAreaHighGrayLevelEmphasis	11792817.97±13233006.64	23078617.40±24225711.24
Column original glszm LargeAreaLowGrayLevelEmphasis	225.22±782.65	184.33±515.83
Column original glszm LowGrayLevelZoneEmphasis	0.01±0.02	0.02±0.02
Column original glszm SizeZoneNonUniformity	168.14±131.85	98.57±72.12
Column original glszm SizeZoneNonUniformityNormalized	0.39±0.08	0.37±0.07
Column original glszm SmallAreaEmphasis	0.65±0.07	0.62±0.06
Column original glszm SmallAreaHighGrayLevelEmphasis	471.48±296.28	362.29±238.24
Column original glszm SmallAreaLowGrayLevelEmphasis	0.01±0.01	0.01±0.01
Column original glszm ZoneEntropy	5.97±0.59	5.55±0.59
Column original glszm ZonePercentage	0.10±0.06	0.06±0.04
Column original glszm ZoneVariance	20161.59±35614.86	34530.68±37097.56
Column original ngtdm Busyness	0.92±2.08	0.95±1.50
Column original ngtdm Coarseness	0.00±0.00	0.00±0.00
Column original ngtdm Complexity	1103.44±926.66	501.48±540.59
Column original ngtdm Contrast	0.08±0.15	0.02±0.04
Column original ngtdm Strength	5.50±3.81	4.32±3.72

2 Supplementary Figures

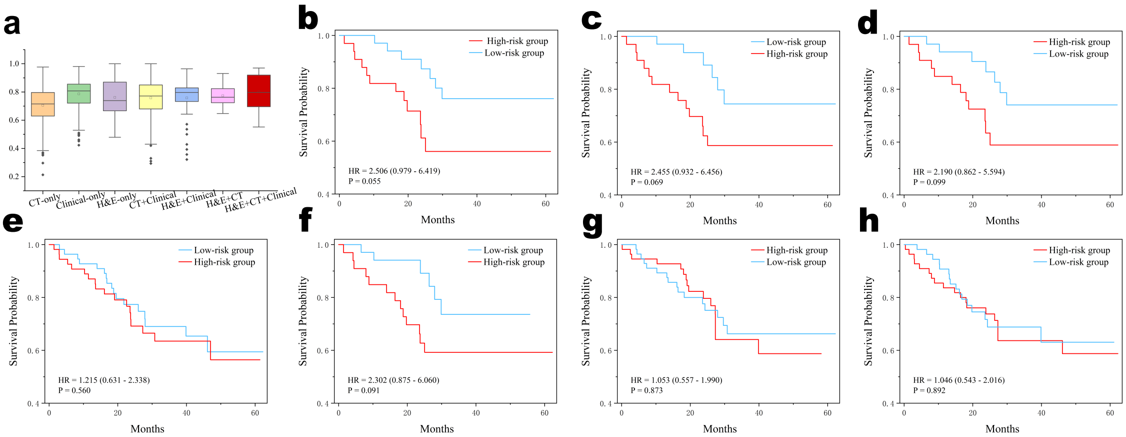

Figure S1: Multimodal features for prediction of overall survival (OS). (a) AUC values in prediction of high-risk (OS<12 months) and low-risk (OS≥12 months) patients of the PD-L1 cohort. Note that statistical significance are detailed in the Supplementary Table 1. (b) Kaplan-Meier curves for high/low risk groups of patients using the H&E + Clinical + CT model. (c) H&E + Clinical, (d) H&E + CT, (e) CT + Clinical, (f) H&E-only, (g) Clinical-only, and (h) CT-only.

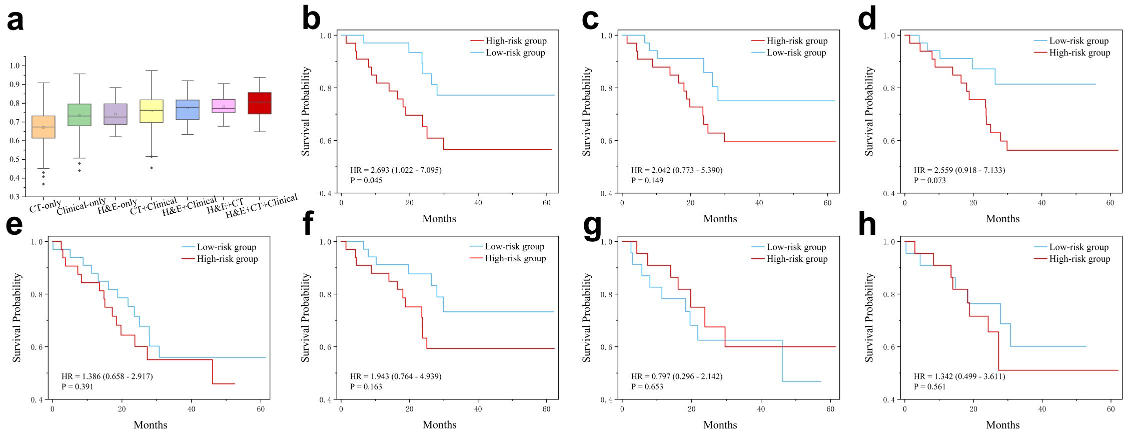

Figure S2: Multimodal features for prediction of overall survival (OS). (a) AUC values in prediction of high-risk (OS<36 months) and low-risk (OS≥36 months) patients of the PD-L1 cohort. Note that statistical significance are detailed in the Supplementary Table 2. (b) Kaplan-Meier curves for high/low risk groups of patients using the H&E + Clinical + CT model. (c) H&E + Clinical, (d) H&E + CT, (e) CT + Clinical, (f) H&E-only, (g) Clinical-only, and (h) CT-only.

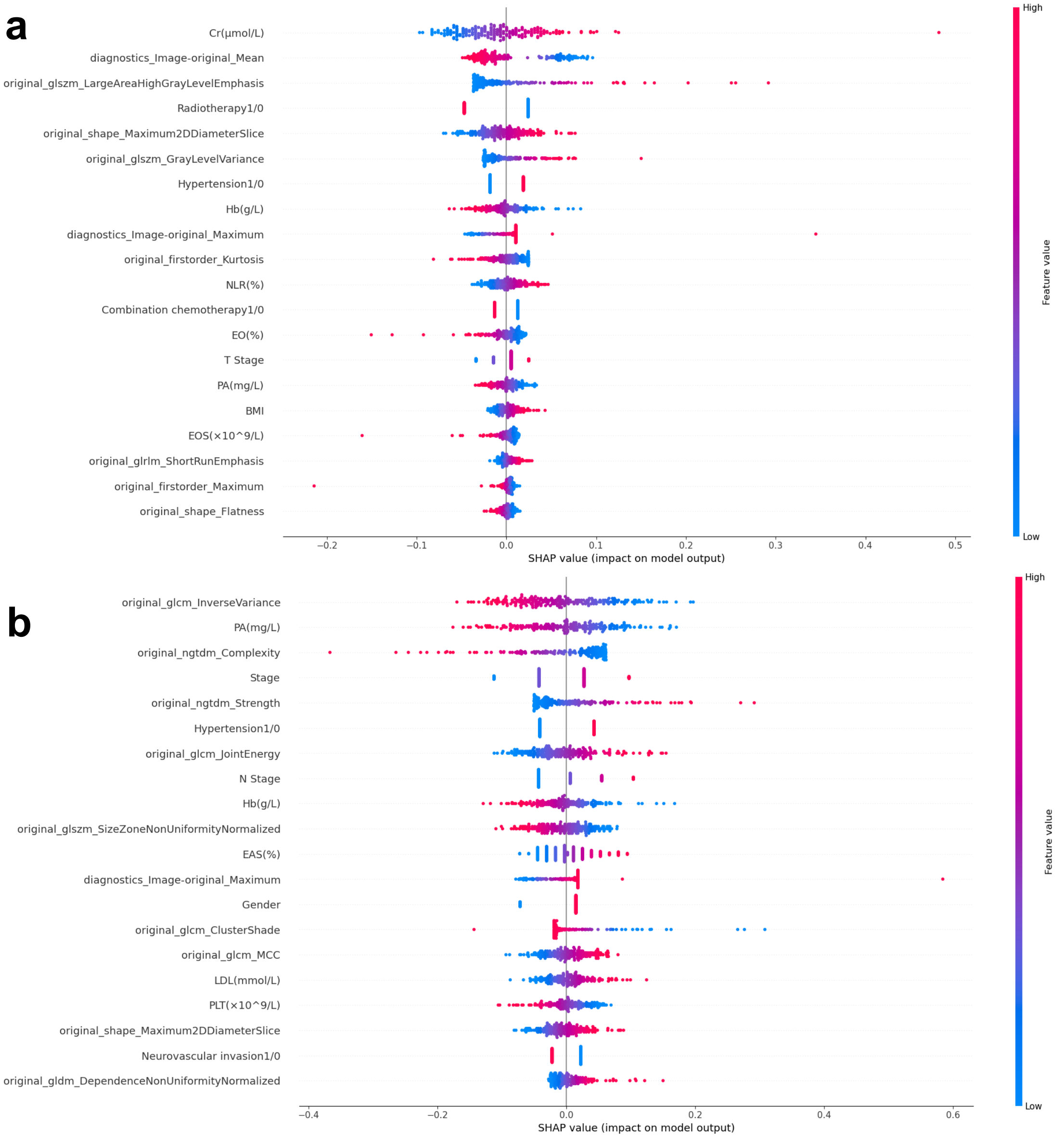

Figure S3: Top features ranked by SHAP values for prognosis. The features are ranked by the sum of SHAP value magnitudes over all samples. (a-b) Top features ranked by SHAP values for predicting high- and low-risk patients using 12-month and 36-month thresholds, respectively.

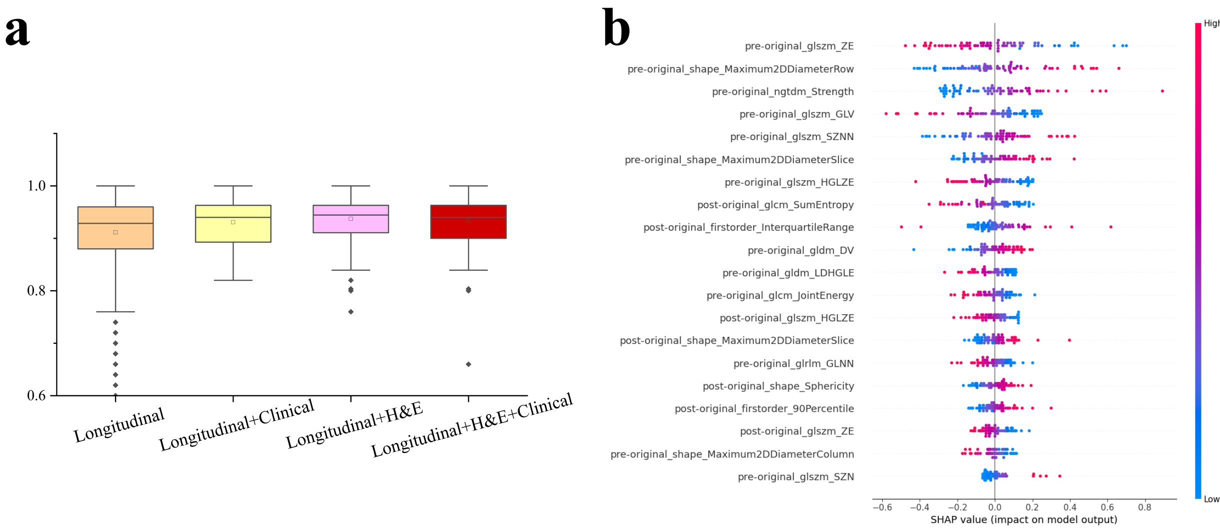

Figure S4: Integration of early on-treatment CT scans further enhanced prediction performance of immunotherapy response. (a) AUC values for predicting responders and non-responders achieved by multimodal and ablated models after integration of longitudinal CT scans. Statistically significant differences are detailed in the Supplementary Table 5. (b) Top 20 radiomic features ranked by SHAP values for predicting immunotherapy response.
